# A novel mycobacterial *In Vitro* infection assay identifies differences of induced macrophage apoptosis between CD4^+^ and CD8^+^ T cells

**DOI:** 10.1371/journal.pone.0171817

**Published:** 2017-02-15

**Authors:** Vanesa Nkwouano, Sven Witkowski, Nidja Rehberg, Rainer Kalscheuer, Norman Nausch, Ertan Mayatepek, Marc Jacobsen

**Affiliations:** 1 Department of General Pediatrics, Neonatology, and Pediatric Cardiology, University Children’s Hospital, Heinrich Heine University, Duesseldorf, Germany; 2 Institute for Pharmaceutical Biology and Biotechnology, Heinrich-Heine-University, Duesseldorf, Germany; National Institute of Infectious Diseases, JAPAN

## Abstract

Macrophages are natural host cells for pathogenic mycobacteria, like *Mycobacterium tuberculosis (M*.*tb)*. Immune surveillance by T cells and interaction with *M*.*tb* infected macrophages is crucial for protection against *M*.*tb* reactivation and development of active tuberculosis. Several factors play a role in the control of *M*.*tb* infection but reliable biomarkers remain elusive. One major obstacle is the absence of functional *in vitro* assays which allow concomitant determination of i) mycobacterial eradication; ii) cytotoxic effects on host macrophages; and iii) effector T-cell functions. We established a novel functional *in vitro* assay based on flow cytometry analysis of monocyte-derived macrophages (MDM) infected with a *Mycobacterium bovis* BCG strain containing a tetracycline inducible live/dead reporter plasmid (LD-BCG). MDM of healthy human donors were generated *in vitro* and infected with defined LD-BCG numbers. After short-term MDM/LD-BCG co-incubation with autologous effector T cells or in the presence of antibiotics, proportions of MDM containing live or dead LD-BCG were determined by flow cytometry. Concomitant measure of defined numbers of added beads allowed comparison of absolute MDM numbers between samples. Differential effects of T-cell subpopulations on anti-mycobacterial cytotoxicity and on MDM apoptosis were determined. Flow cytometry measure of MDM/LD-BCG treated with rifampicin correlated well with mycobacterial colony forming units and fluorescence microscopy results. Co-culture with pre-activated effector T cells reduced viability of both, LD-BCG and MDM, in a concentration-dependent manner. *M*.*tb* protein specific CD4^+^ and CD8^+^ T-cells contributed similarly to anti-mycobacterial cytotoxicity but CD4^+^ T cells induced higher levels of apoptosis in infected MDMs. This novel assay enables rapid quantification of anti-mycobacterial cytotoxicity and characterization of effector functions. Our functional *in vitro* assay has the potential to contribute to the identification of biomarkers for protective T-cell responses against tuberculosis.

## Introduction

Tuberculosis (TB) is a major public health issue with worldwide incidence of about nine million cases and estimated two billion *Mycobacterium tuberculosis* infected individuals [1]. Immune surveillance is central for protection against progression to active disease of about 90% of *M*.*tb* infected individuals but reliable prediction of TB susceptibility remains elusive. The interaction of alveolar macrophages–the predominant host cell of *M*.*tb*–and effector T cells is decisive for the outcome of infection. Multifaceted mechanisms are involved in this process including, i) activation of macrophage effector functions e.g. by T cells producing IFNγ and TNFα, ii) reversal of *M*.*tb* induced endosome maturation blockade, iii) inhibition of mycobacterial growth, and iv) T cell-mediated killing of intracellular *M*.*tb* as well as, eventually, infected host macrophages [reviewed in [2]]. It is a matter of debate whether killing of macrophages is beneficial or detrimental, since macrophage death is not necessarily accompanied by killing of intracellular mycobacteria [3]. The pathway of cytotoxicity is decisive and there is compelling evidence that exclusively apoptotic cell death together with certain effector molecules (i.e. granulysin) is able to kill mycobacteria [4, 5]. Against this background of complex and fine-tuned immune effector mechanisms, we assumed that methods applied to test novel candidates of host/pathogen interaction need to incorporate this complexity. In addition it may be informative to concomitantly characterize macrophages/T cell interaction as part of ‘functional assays’.

So far functional anti-mycobacterial assays are previously mainly based on mycobacterial growth inhibition determined by mycobacterial culture e.g. by colony-forming units (CFU) [6–10]. CFU analysis is laborious (about two to three weeks) and solely focuses on mycobacterial growth whereas concomitant analyses of host immune cells and effector molecules are not possible. In an attempt to include immune effector molecules in functional anti-mycobacterial assays, Wallis et al. established a method that enabled to determine the effect of antibiotic treatment and host immune cells concomitantly using a whole blood based approach [11]. This assay predicted the efficacy of anti-tuberculosis chemotherapy [12] and BCG vaccination as well as revaccination [13]. The group of Kampmann et al. greatly promoted the progress of functional assay development by introducing a *M*. *bovis* BCG reporter strain (i.e. BCG-*lux*) to monitor the mycobacterial growth in human whole blood cultures [14, 15]. This comprised three major advantages i) incorporation of the majority of immune populations involved in anti-mycobacterial host response; ii) rapid luminescence-based readout to avoid long-term mycobacterial culture; iii) avoidance of sophisticated immune cell purification techniques to assure broad applicability. Furthermore the usage of *M*. *bovis* BCG allowed the application also in biosafety level (BSL)-1 facilities. Subsequent studies modified the BCG-lux assay to analyse effects of cytokines during *M*.*tb* infection [16], immune responses to BCG vaccination [17], and the effect of vitamin D supplementation on anti-mycobacterial immunity [18]. Mycobacterial fluorescent reporter strains have rarely been used to determine viability due to the long half-live of fluorescent proteins. To circumvent this obstacle Martin *et al*. generated a *M*.*tb* H37Rv strain containing a live-dead reporter plasmid [19]. The live-dead H37Rv strain constitutively expresses mCherry and–on induction by tetracycline derivatives (such as anhydrotetracycline (ATC))–concomitantly expresses GFP. This strain enabled quantification of live and dead mycobacteria inside macrophages and results strongly correlated with mycobacterial culture method [19].

In the present study we applied multi-colour flow cytometry to establish an *in vitro* assay of mycobacterial host interactions based on a live-dead *M*. *bovis BCG* (LD-BCG) infection. This assay enabled us to exactly quantify mycobacteria growth inhibition by antibiotics as well as effector T cells in a short-term (about 24h) co-culture assay. Concomitantly viability and phenotype of infected monocyte-derived macrophages (MDMs) and effector T cells were determined. In addition the functional relevance of cytotoxic mechanisms in T-cell subpopulations were evaluated. To our knowledge this is the first mycobacterial infection assay that allows quantification of anti-mycobacterial cytotoxicity and characterization of involved functional mechanisms concomitantly.

## Results

### A live-dead reporter BCG strain (LD-BCG) allowed quantification of infected MDM by flow cytometry

**Fig 1:**
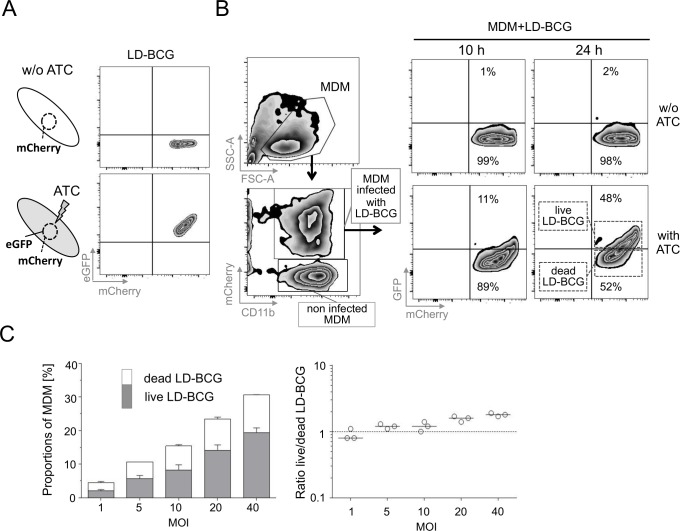
Optimization of MDM infection with live/dead (LD)-BCG and tetracycline (ATC) induced GFP expression. (A) LD-BCG analysis by flow cytometry. Schematic depiction of LD-BCG (left) and flow cytometry analysis of LD-BCG with ATC (upper panel) or without (lower panel) are shown as zebra plots. (B) Flow cytometry analysis of MDM after LD-BCG infection. Gating procedure of MDM (left graphs) and ATC induced GFP expression after different time periods of MDM infection are shown. Black arrows depict sequence of analysis steps (C) MDM with LD-BCG at different Multiplicity Of Infection (MOI). Proportions of MDM infected with live (grey) and dead (open) LD-BCG are indicated by stacked box plots (left graph) and as ratios of LD-BCG (live divided by dead) in MDM. Median values with range of triplicates are depicted. A representative experiment of five is shown.

Mycobacterial reporter strains expressing fluorescent proteins can be used for flow cytometry-based analysis of monocyte-derived macrophage (MDM) infection. The fluorescent live-dead reporter *M*.*tb* strain H37Rv, described by Martin *et al*. [19], allowed determination of mycobacterial viability. We used the same construct to generate a live-dead *M*. *bovis BCG* reporter strain (LD-BCG) that permanently expresses mCherry and, if bacteria are viable, can be induced to co-express GFP by the tetracycline derivative ATC. ATC induced GFP in the vast majority of mCherry positive LD-BCG indicating high proportions of live mycobacteria in culture (Fig 1A). ATC treatment of MDMs infected with LD-BCG induced GFP only in a subset of infected MDMs (Fig 1B). This could be due to decreased viability or suboptimal conditions for ATC mediated induction. To test this, we added ATC for different co-culture periods (i.e. 10 h; 24 h) and detected markedly higher proportions of GFP positive infected MDMs after 24 h (48%) as compared to 10 h (11%) (Fig 1B). Therefore, both, suboptimal ATC-induced GFP induction and MDM mediated anti-mycobacterial cytotoxicity contributed to reduced proportions of GFP-positive MDM infected with LD-BCG. To optimize the LD-BCG multiplicity of infection (MOI), we applied different LD-BCG concentrations and detected increased MDM infection rates at higher MOI levels (Fig 1C, left graph). At a MOI of 20:1 about 25% of MDM were infected with LD-BCG. In addition the relative proportion of live LD-BCG increased at higher MOIs and reached a plateau at an MOI of 20:1 (Fig 1C, right graph). Accordingly we applied an MOI of 20:1 for further experiments.

### Flow cytometry based quantification of LD-BCG infected MDM and CFU showed comparable results

**Fig 2:**
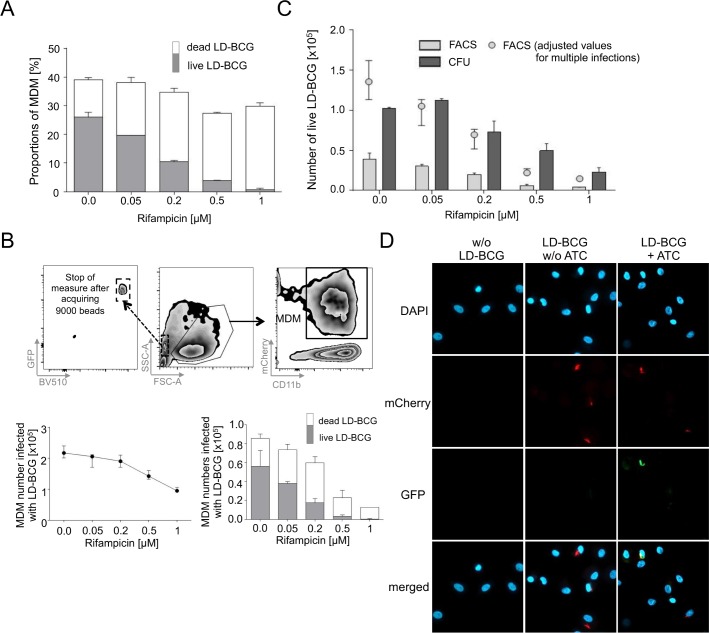
Proportional and absolute differences of LD-BCG infected MDM and comparison with Colony Forming Units (CFU) and fluorescence microscopy. Rifampicin treatment of MDM infected with LD-BCG at different indicated concentrations is shown. (A) Flow cytometry (FACS) analysis of MDM proportions infected with live (grey) or dead (open) LD-BCG are shown as stacked boxes. (B) Comparison of absolute MDM numbers infected with LD-BCG measured by bead corrected flow cytometry. Bead-based standardization of measured sample volume is shown in the upper graphs. Absolute numbers of all infected MDM (lower left graph) as well as MDM containing live and dead LD-BCG (lower right graph) at different rifampicin concentrations are shown. Median with range of triplicates are depicted. (C) Comparison of live LD-BCG infected MDM numbers measured by FACS and mycobacterial culture (CFU). Circles indicate FACS values adjusted for multiple infections as determined by fluorescence microscopy. Median values with range of triplicates are depicted. (D) Fluorescence microscopy analyses of MDMs infected with LD-BCG with or w/o ATC or non-infected MDM are shown. Blue color indicates MDM nuclei; red color indicates mCherry expressing LD-BCG; green color indicates GFP-expressing live LD-BCG. A representative experiment of three is shown.

Rifampicin mediated LD-BCG eradication was used to evaluate anti-mycobacterial efficacy and possible side effects on MDM. Increasing concentrations of rifampicin steadily reduced the proportion of MDM containing live LD-BCG whereas MDM with dead LD-BCG increased (Fig 2A). At higher rifampicin concentrations (0.5 – 1 μM) the proportion of MDM infected with dead LD-BCG remained relatively stable. As this could be due to rifampicin-mediated cytotoxic side effects on MDM, we analysed absolute numbers of MDM. This approach required standardization of sample acquisition by flow cytometry. In this regard we applied fluorescent bead counts to analyse comparable sample volumes by flow cytometry (Fig 2B, upper plots). MDM numbers were similar at low concentrations (0.02–0.2 μM) and without rifampicin (Fig 2B, lower left graph). However at higher rifampicin concentrations (0.5 – 1 μM) diminished MDM numbers were detected per sample indicating cytotoxic rifampicin effects on MDM (Fig 2B, lower left graph). Direct evaluation of absolute MDM counts showed decreased numbers of MDM infected with LD-BCG (mainly due to decreased numbers of MDM containing live LD-BCG) whereas the number of MDM containing dead LD-BCG are slightly increasing at low rifampicin concentrations (Fig 2B, lower right graph). At high rifampicin concentrations, very low levels of live LD-BCG containing MDM were detectable and also the number of dead LD-BCG containing MDM was decreased (Fig 2B, lower right graph). We concluded that our novel assay allows for discrimination between proportional and absolute differences of MDM infected with live or dead LD-BCG. In addition, anti-mycobacterial killing with or without concomitant cytotoxicity against MDM can be characterized.

To determine the reliability of flow cytometry-based quantification of LD-BCG viability, we compared live LD-BCG infected MDM numbers with mycobacterial culture growth measured by colony forming units (CFU) from rifampicin-treated cultures. CFU and MDM infected with live LD-BCG correlated strongly (r = 0.84, p < 0.01) (data not shown) but CFU counts were at average higher as compared to flow cytometry-based prediction at corresponding rifampicin concentrations (Fig 2C). Multiple LD-BCG infections of single MDM may account for these differences and, therefore, we determined the number of live LD-BCG per MDM by fluorescence microscopy (Fig 2D). LD-BCG infected MDM had a mean infection rate of 3.4 (SD = 0.7) and adjustment for this factor suggested that multiple infections accounted for differences between CFU and flow cytometry data (Fig 2C, grey circles). We concluded that flow cytometry analysis reliably predicted viability of LD-BCG and that adjustment for multiple MDM infection revealed comparable results to CFU.

### *M*.*tb*-specific and polyclonal activated effector T cells reduce LD-BCG and MDM viability

**Fig 3:**
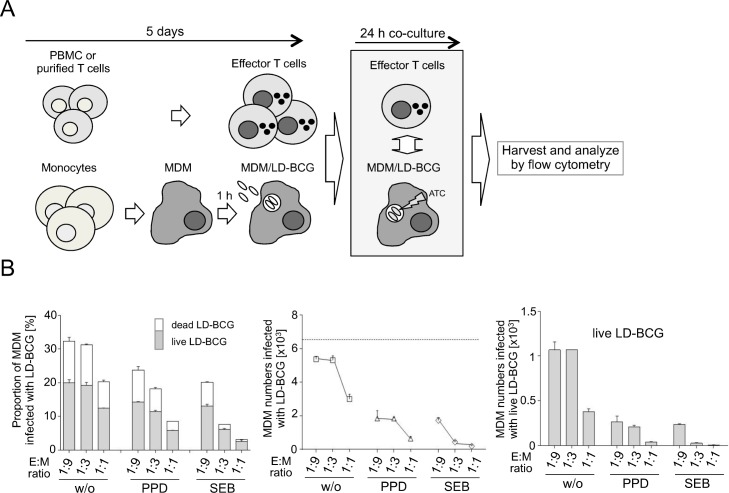
*In vitro* generation of effector T cells (E) and co-culture with LD-BCG infected MDM (M) at different E/M ratios. (A) Workflow depiction for the generation of effector T cells, LD-BCG infected MDM, E/M co-culture, flow cytometry analyses of infected MDM and effector T cells. (B) Analyses of MDM infected with LD-BCG after co-culture with effector T cells stimulated with *Staphylococcus* Enterotoxin B (SEB), *M*. *tuberculosis* Purified Protein Derivative (PPD), and without stimulation (w/o). Different E/M ratios are shown on the x-axes. Proportions of infected MDM with live (grey) or dead LD-BCG (open) are shown as stacked boxes (left graph). Absolute numbers of infected MDM are shown as symbols (middle graph) and numbers of MDM infected with live LD-BCG are shown as boxes (right graph). The dotted line in the middle graph indicates MDM numbers infected with LD-BCG w/o effector T-cell co-culture. Median with range of triplicates from a representative experiment are depicted.

To analyse the influence of effector T cells on MDM infected with LD-BCG, we stimulated PBMC of healthy donors *in vitro* and co-cultured autologous effector T cells and infected MDM at different effector/target (E:M) ratios. Effector T cells were stimulated with *M*.*tb* antigen (i.e. PPD) or the polyclonal T-cell activator staphylococcus enterotoxin B (SEB). Fig 3A depicts the experimental procedure and Fig 3B shows results of a representative experiment. The highest proportions of infected MDMs were detected with non-stimulated T cells (Fig 3B, left graph). Increasing effector T cell numbers (higher E:M ratios) resulted in decreased proportions of live LD-BCG infected MDM for non-stimulated and *in vitro* activated ‘effector’ T cells (Fig 3B, left graph). Effector T cells also reduced MDM numbers in a concentration and stimuli-dependent manner (Fig 3B, middle graph). This resulted in markedly reduced MDM numbers by polyclonal activated or mycobacteria specific effector T cells as compared to non-stimulated T cells or infected MDM w/o effector T cells (Fig 3B, middle graph). MDM numbers infected with live LD-BCG (Fig 3B, right graph) revealed marked differences between *in vitro* activated and non-stimulated T cells.

**Fig 4:**
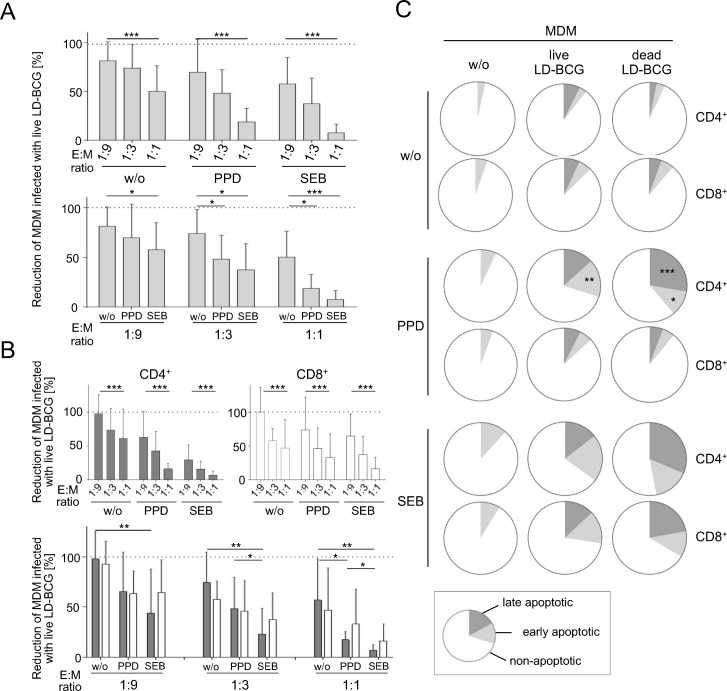
Cytotoxic effects of total effector T cells and T-cell subpopulations against LD-BCG infected MDM and markers of induced cell death on MDM. Combined analyses of autologues E/M samples from healthy donors (n = 8) for (A), (n = 6) for (B), and (n = 5) for (C) are shown. Total effector T cells (A) or enriched CD4^+^/CD8^+^ T-cell subpopulations (B, C) were applied. (A, B) Cytotoxicity of differentially stimulated effector T cells against MDM infected with LD-BCG was assessed by flow cytometry. Different E/M ratios were applied (x-axes) and absolute numbers of MDM infected with live LD-BCG are indicated (y-axes). Mean and standard deviations are shown. (C) Apoptosis marker expression on MDM with or w/o LD-BCG infection after coculture with differentially stimulated CD4^+^ or CD8^+^ effector T cells. E:M ratios of 1:3 are shown. The proportions of non-apoptotic (open), early apoptotic (bright grey), and late apoptotic (dark grey) MDM are depicted as pie charts. Non-infected MDM (left panel) as well as MDM infected with live (middle) and dead (left) LD-BCG are shown. Median values of five independent experiments are depicted. The Mann-Whitney U-test were applied. Asterisks indicate significant differences (***: p<0.001; **: p<0.01; *: p<0.05).

Combined analyses of independent experiments using MDM/effector T cells from different donors require correction for absolute numbers of MDM infected with LD-BCG. Therefore the number of LD-BCG infected MDMs–without effector T cells–was set to 100% and the relative differences (≅ cytotoxic efficacy) for each effector T-cell population were calculated. Comparisons of cytotoxic efficacy of differentially stimulated effector T cells from healthy donors (n = 8) are shown in Fig 4A. Effector T cells showed E:M ratio dependent anti-mycobacterial cytotoxic efficacy (ANOVA, p < 0.001 for all stimuli) (Fig 4A, upper graph). In addition significantly higher cytotoxic efficacy after SEB induced polyclonal T-cell activation (p = 0.04, p = 0.02, p<0.001 for 1:9, 1:3, 1:1, respectively) as well as PPD-specific stimulation (for 1:3, p = 0.04; for 1:1 p = 0.02) was detected, compared to non-stimulated samples (Fig 4A, lower graph). Therefore E:M ratio as well as stimulation dependent differences in cytotoxic efficacy of T cells significantly affected viability of LD-BCG infected MDMs. Since stimulated effector cells from PBMC contain different subpopulations of T cells (S1 Fig), we further characterized relevant subpopulations by purifying CD4^+^ and CD8^+^ T cells before *in vitro* culture.

### CD4^+^ and CD8^+^ T cells exert comparable cytotoxic effects but differ in PPD induced infected MDM kill mechanisms

CD4^+^ and CD8^+^ T cells were separately stimulated and co-cultured with infected MDM. Both, CD4^+^ or CD8^+^ T cells exerted cytotoxic effects on LD-BCG infected MDMs in a titration dependent manner (ANOVA, p< 0.001 for CD4^+^ as well as CD8^+^ T cells and for all stimuli) (Fig 4B, upper graph). As shown for PBMC, PPD and SEB stimulation induced enhanced cytotoxic efficacy of CD4^+^ T cells (for p-values see Table 1) (Fig 4B, lower graph). The stimuli induced differences for CD8^+^ T cells were not significant (Fig 4B, lower graph and Table 1). Notably, no significant differences were detected between CD4^+^ and CD8^+^ T cells for different stimuli and at any E:M ratio (Table 1).

**Table 1:** Stimulus dependent reduction of MDM with live LD-BCG by co-culture with CD4^+^ and CD8^+^ T cells.

E:M	Comparison	CD4^+^ T cells		CD8^+^ T cells	
		p value	Significance	p value	Significance
	w/o vs PPD	0.093	ns	0.132	ns
1:9	w/o vs SEB	0.002	**	0.132	ns
	PPD vs SEB	0.258	ns	0.999	ns
	w/o vs PPD	0.093	ns	0,387	ns
1:3	w/o vs SEB	0.004	**	0.132	ns
	PPD vs SEB	0.028	*	0.571	ns
	w/o vs PPD	0.015	*	0.571	ns
1:1	w/o vs SEB	0.002	**	0.132	ns
	PPD vs SEB	0.017	*	0.474	ns

Mann-Whitney U-test results are indicated; E:M: Effector T-cell/MDM ratio; ns: not significant

*: p<0.05

**: p<0.01

A central T-cell dependent anti-mycobacterial effector function is the induction of apoptosis in infected macrophages. Therefore we determined the expression of early and late apoptotic markers in infected and non-infected MDMs after co-culture. Co-culture with non-stimulated CD4^+^ or CD8^+^ T cells induced only marginal expression of early and late apoptotic markers in MDMs infected with live or dead LD-BCG (Fig 4C, upper panel). But whereas SEB stimulated CD4^+^ and CD8^+^ T cells induced increased expression of early and late apoptotic markers in MDM (Fig 4C, lower panel; Table 2), only PPD specific CD4^+^ effector T cells (Fig 4C, middle panel) induced increased expression of early apoptosis markers in MDM infected with live (p = 0.003) and dead LD-BCG (p = 0.01) and increased late apoptotic markers in MDM infected with dead LD-BCG (p<0.001) as compared to CD8^+^ effector T cells. We concluded that PPD induced comparable cytotoxic efficacy of CD4^+^ and CD8^+^ T cells against LD-BCG infected MDM, but cytotoxic mechanisms differed between T-cell subsets with regard to apoptosis induction. We concluded that flow cytometry-based characterization of effector T-cell/MDM interaction in mycobacterial infection can elucidate relevant functional differences, not detectable in conventional viability assays.

**Table 2:** Comparison of expression of apoptotic markers induced by differentially activated CD4^+^ and CD8^+^ T cells in MDM with live BCG (E:M 1:1)

	Comparison	Apoptosis	CD4^+^ T cells		CD8^+^ T cells	
			p-value	Significance	p-value	Significance
live LD-BCG	w/o vs PPD	early	<0.001	***	0.99	ns
		late	0.08	ns	0.86	ns
	w/o vs SEB	early	<0.001	***	0.004	**
		late	0.25	ns	0.25	ns
	PPD vs. SEB	early	0.21	ns	0.007	**
		late	0.90	ns	0.16	ns
dead LD-BCG	w/o vs PPD	early	0.006	**	0.92	ns
		late	<0.001	***	0.29	ns
	w/o vs SEB	early	0.001	**	0.08	ns
		late	<0.001	***	<0.001	***
	PPD vs. SEB	early	0.39	ns	0.07	ns
		late	0.43	ns	0.001	**

Mann-Whitney U-test results are indicated; ns: not significant

**: p<0.01

***: p<0.001

## Discussion

Functional *in vitro* assays are important tools to evaluate the relevance of immune biomarkers in mycobacterial infections. The present assay extends the potential of previous functional assays by characterizing the immune cell interplay and possible harmful side effects on mycobacteria hosting macrophages. The application of flow cytometry to detect a fluorescent BCG reporter strain allows rapid detection of live and dead mycobacteria within MDM and circumvents the need for long-term mycobacteria culture. Standardization of flow cytometry measures allows absolute quantification of surviving and killed MDM with or without mycobacterial infections. Concomitant characterization of immune cell phenotype is possible to compare e.g. cytotoxic mechanisms as performed for apoptosis in the present study.

We generated a BCG fluorescent reporter strain based on a previous publication [19]. The usage of the BCG vaccine strain permits implementation of this assay in the majority of facilities including hospitals and research institutions in low-income countries whereas the use of virulent *M*.*tb* strains requires biosafety level 3 facilities, which are rarely available. BCG mycobacteria do not contain the virulence associated RD1 region of *M*.*tb*. As a consequence maturation blockade of early phagosomes–a typical feature of MDM infected with virulent *M*.*tb* [2]–is less prevalent in BCG. In accordance MDM infected with LD-BCG alone (without rifampicin or activated effector T cells) reduced the viability of mycobacteria in an MOI associated manner (Fig 1B). However, further reduction of viability was dependent on MDM co-culture with activated T cells or antibiotic treatment. Future studies will determine if the results of the LD-BCG based MDM kill assay reflect the immune response in the context of *M*.*tb* MDM infection.

We demonstrated that LD-BCG viability in MDM (indicated by GFP expression) strongly correlates with mycobacterial culture measured CFU, the conventional method to determine mycobacterial viability. The comparison of bead-normalized numbers of MDMs infected with live LD-BCG revealed lower numbers compared to CFU (Fig 2C). Three reasons may account for this. First, ATC-induced GFP expression of LD-BCG within MDM may be suboptimal since longer incubation increased GFP expression (Fig 1B) whereas extracellular LD-BCG showed discriminant GFP expression in the presence of ATC (Fig 1A). Therefore not all LD-BCG may respond to ATC when engulfed by MDM and the calculated proportions/absolute numbers of live LD-BCG containing MDM may be underestimated. General reduced viability of LD-BCG in MDM (accompanied by decreased GFP expression) confounds estimation of this effect. Second, MDM inherent background fluorescence in the GFP channel (especially detected in mCherry_high_ MDM not treated with ATC) limited the detection sensitivity for live LD-BCG in MDM. Initial FACSort experiments to purify MDM with different levels of GFP expression (data not shown) support the notion that one of these explanations affects analyses. Third, multiple LD-BCG infection of single MDM may account for differences between flow cytometry and CFU based calculation. Measurement of single MDM mycobacterial burden by fluorescence microscopy revealed multiple infections per cell (here a mean infection rate of 3.4-fold). Therefore the effect of multiple LD-BCG infection of single MDMs largely accounts for differences between flow cytometry and CFU. Although adjustment for multiple infections is theoretically possible on the basis of flow cytometry data, MDM background fluorescence and variability in LD-BCG fluorescence intensity render this approach error-prone. Therefore the sensitivity of the flow cytometry assay for detection of LD-BCG is about 3-times lower as compared to CFU.

The stability of the assay is substantiated by including different rifampicin concentrations or effector cell numbers as well as triplicates within individual experiments. Steady anti-correlation of decreased MDM/LD-BCG numbers/proportions with increased rifampicin concentrations or E:T ratios was detected throughout the experiments and confirm stability. In addition minor standard deviations of triplicates shown for representative experiments in Figs 1 to 3 indicate stability.

In contrast MDM/LD-BCG infection rate and mediated cytotoxicity of effector T cells varied markedly between experiments using PBMC from different donors. To enable concomitant depiction of experiments from different individuals we set the number of MDM infected with LD-BCG to 100% and calculated the proportional reduction induced by different effector T cells. We termed this measure ‘cytotoxic efficacy’ and depicted values in Fig 4. The ‘cytotoxic efficacy’ varied between effector cells from different donors and high standard deviations (Fig 4A and 4B) indicate this. However, paired statistical tests verify significant differences of different stimuli and E:T ratios from independent experiments. The marked standard deviations in Fig 4 are therefore not due to weak assay stability but reflect differential cytotoxic efficacy of individual donor effector T cells. Variability in the T-cell response against SEB or PPD can be causative as well as different maturation stages of T-cell populations *in vivo*. Ongoing studies aim at elucidating this question.

Previous functional mycobacterial *in vitro* assays focussed on quantification of pathogen viability [6–10], but characterization of host monocytes/macrophages and effector T cells concomitantly has not been addressed. The novel assay allows i) characterization of MDM infected with live or dead LD-BCG as well as without infection, ii) absolute and/or proportional quantification of MDM after co-culture to determine the influence of sample conditions on host MDM viability and infection status, iii) analysis of functional activities of effector T-cell populations e.g. in the context of anti-mycobacterial killing efficiency, and iv) analysis of anti-mycobacterial effects of novel immune cell molecules or drug candidates. Discrimination between MDM infected with live and/or dead bacteria, as well as non-infected cells renders identification of phenotypic changes of MDM relevant for effective mycobacterial eradication possible. In this way we detected increased proportions of MDM showing apoptosis-associated markers induced by PPD-specific CD4^+^ T cells that were not detected for PPD-specific CD8^+^ T cells. This is in accordance with previous findings that apoptosis is crucial for mycobacterial killing [20]. For CD4^+^ T cells, Fas ligand induced apoptosis was shown to be the predominant effector mechanism against *M*.*tb* [21] and regulation of Fas ligand expression on T cells e.g. by IFNγ may account for differential capacity to induce apoptosis [22]. Ongoing studies will determine if differences of induced apoptosis correlate with differential FasL expression on T-cell subpopulations. In addition our results rendered a role of direct cytotoxic effects of CD8^+^ effector T cells [5] and/or cell death-independent macrophage effector mechanisms (e.g. autophagy, efferocytosis) likely [19, 23].

Different effector cell populations and T-cell stimulations can be compared for their cytotoxic and anti-mycobacterial effects. Since *in vitro* activation precedes the co-culture, we can largely control for confounding effects like differential proliferation by adjusting cell numbers before co-culture. Multicolour flow cytometry enabled us to characterize effector cell populations concomitantly. Identification of effector cell mechanisms associated e.g. with mycobacterial killing or MDM viability is therefore feasible. As an initial approach we purified CD4^+^ and CD8^+^ T cells before *in vitro* stimulation and showed that both populations exert anti-mycobacterial killing but that effector mechanism may differ.

This functional *in vitro* assay can be considered for bridging between descriptive patient studies and animal models to identify relevant biomarkers for host immunity against mycobacterial infections.

## Material and methods

### Ethics statements

This study was approved by the ethics committee of the University Hospital Duesseldorf (Internal Study No. 4505). Written informed consent was obtained from all participating donors.

### Isolation of Peripheral Blood Mononuclear Cells (PBMC) and purification of CD4^+^ and CD8^+^ T cells

Buffy coats (about 40 ml) from healthy donors were purchased from the Institute for Transfusion Medicine of the University Hospital Duesseldorf. PBMC were isolated by density gradient using Biocoll Separating Solution (Biochrom) following manufacturer´s instructions. The average cell number was between 0.7 to 1 x 10^9^ PBMC. CD4^+^ and CD8^+^ T cells were enriched by magnetic cell sorting (IMag, BD Biosciences) of freshly isolated PBMC using magnetic beads labelled with CD4 or CD8 specific antibodies (BD Biosciences) following manufacturer´s instructions. The purity of enriched cells was > 95% as assessed by flow cytometry.

### Generation of effector cells

Freshly isolated PBMC or enriched CD4^+^ and CD8^+^ cells were cultured in round-bottomed 96-well plates (Greiner) at 1.5 x 10^5^ cells/well in RPMI 1640 (Gibco) supplemented with 1% L-Glutamine (Sigma-Aldrich), 10% heat inactivated human AB serum (Sigma-Aldrich) and 10 mM HEPES (Lonza) (for simplicity reasons called complete medium in the remaining manuscript) for 5 days at 37°C and 5% CO_2_. Cells were stimulated with purified protein derivatives of *M*.*tb*. (PPD; 10 μg/ml; Statens Serum Institute), or *Staphylococcus* enterotoxin B (SEB; 1.5 μg/ml; Sigma-Aldrich). Thereafter the cells were washed to remove antigens and counted.

#### Enrichment of monocytes and Monocyte-Derived Macrophage (MDM) differentiation

Autologous monocytes were enriched from freshly isolated PBMC using CD14-labeled magnetic beads using the IMag system (IMag, BD Biosciences) following manufacturer´s instructions. The purity of enriched monocyte populations was > 90% as determined by flow cytometry. Enriched monocytes (1 x 10^5^ cells/well) were cultured in 96 well flat-bottomed plates (Greiner) in 200 μl complete medium for 5 days at 37°C and 5% CO_2_ to generate MDMs. Thereafter the number of live MDMs per well was about 2–3 x 10^4^ as assessed by cell counting.

### Generation and culture of LD-BCG

The Live-Dead (LD) reporter plasmid harbours a gene encoding mCherry fluorescent protein constitutively expressed from the hsp60 promotor, a gene encoding green fluorescent protein (GFP) under the control of a tetracycline-inducible promotor, as well as a hygromycin resistance cassette. It allows differentiation between live and dead mycobacteria [19]. Cells of *M*. *bovis* strain BCG Pasteur were grown to log-phase in Middlebrook 7H9 medium (BD Biosciences) supplemented with 10% ADC (BD Biosciences) and 50 μg/ml hygromycin (Gentaur), electroporated with the LD reporter plasmid at 2.5 kV, 1000 Ω and 25 μF using a Biorad Gene Pulser, and plated on 7H11 agar supplemented with 10% OADC and 50 μg/ml hygromycin for selection. Individual clones were picked after 20 days of incubation, cultured in liquid media, and glycerol stocks were frozen at -80°C. For infection experiments, cryopreserved LD-BCG bacteria were inoculated in 7H9 culture medium and cultured at 37°C and 90 rpm until an OD_600 nm_ of 0.7 to 0.8 was reached.

#### In vitro infection of macrophages with LD-BCG and co-culture with effector T cells

Following 5 days of MDM culture, medium was removed and 100 μl complete medium (pre-warmed to 37°C) was added to the wells. 5 ml of the LD-BCG culture was centrifuged, washed in complete medium, and titre was estimated based on OD-measurement, with an OD_600nm_ = 1 corresponding to 3 x 10^8^ CFU/ml. MDM were infected at different MOIs (multiplicity of infection) by adding the defined number of LD-BCG containing complete medium (100μl per well) or left non-infected by adding complete medium only. After centrifugation at 209 x g for 3 min at room temperature, the cells were incubated for 3 h at 37°C and 5% CO_2_.

LD-BCG-infected MDM cultures were washed with complete medium (pre-warmed to 37°C) to remove extracellular bacteria. Effector cell cultures were washed in 1 ml pre-warmed complete medium and counted in Neubauer cell chambers. Thereafter infected MDMs were incubated with effector cells for 24 h at an effector to MDM (E/M) ratio of 1:9, 1:3 or 1:1 in a final volume of 200 μl/well. Anhydrotetracycline hydrochloride (ATC, 0.2 μg/ml final concentration; Sigma-Aldrich) was added for induction of GFP expression in live LD-BCG.

### Cell surface staining and FACS analysis

After infection of MDM and co-culture with effector cells, cells were washed in PBS containing 10 mM EDTA and 0.5% BSA (washing medium; on ice), the supernatant was discarded, and the cells were stained with antibodies against CD11b (PE-Cy7, clone ICRF44, BioLegend) and viability dye eFluor 780 (eBioscience) in the residual volume for 30 min on ice. After washing in washing medium, cells were resuspended and directly measured by flow cytometry or, for cell death analyses, cells were stained for Annexin V (BV421; BioLegend) and 7-AAD (BioLegend) according to manufacturer’s instructions. Flow cytometry measurements were performed using a LSR Fortessa flow cytometer (BD Biosciences). Before measurement, 123-count eBeads (10 μl; stock: 1000 beads/μl; eBioscience) of were added to each sample. Flow cytometry measure stopped automatically when 9000 beads were acquired. FlowJo software (Version 10; Tree Star) was used for data analysis. Analyses details and gating procedure is indicated as part of Figs 1, 2, and 3. ATC-independent GFP expression was subtracted from each individual sample.

To determine the absolute numbers of MDM infected with live or dead LD-BCG, the absolute number of MDM of each sample was multiplied by the respective proportion of infected MDM with live or dead LD-BCG.

For inter-individual comparisons of anti-mycobacterial T cell responses, the number of MDM infected with live or dead BCG was set to 100% for the sample with MDM infected with LD-BCG alone (without activated effector T cells) in each experiment, and the relative change was calculated for each of the effector cell-treated samples within the experiment.

#### Determination of Colony Forming Units (CFU)

Following infection of MDM and co-culture with effector cells, cells were centrifuged (800 x g for 5 min) and supernatant was removed. 120 μl sterile PBS containing 0.5% Tween80 (Sigma-Aldrich) was added to lyse MDMs. The plates were then incubated for 30 min on ice and serial dilutions were performed in PBS / 0.5% Tween. 50 μl of each dilution were plated on 7H11 agar supplemented with 10% OADC and 50 μg/ml hygromycin. CFU were counted after 14 to 18 days of incubation at 37°C.

### Microscopic analysis

Cell culture (generation of MDMs and effector cell co-culture) was performed in 8-well Permanox®-mounted chamber slides (Lab-Tek). Thereafter medium was removed, cells were fixed with fixation buffer (50μl/well; BioLegend) at room temperature for 15 min, and washed with PBS (Gibco). After incubation with serum-free Protein Block buffer (Dako) for 15 min at RT, cell nuclei were stained with DAPI Dihydrochloride (Calbiochem) following manufacturer’s instructions. Then mounting medium was added and cells were covered with cover glass. An Axio Observer.Z1 fluorescent microscope (Carl Zeiss) with ZEN Pro Software (Carl Zeiss) was used for imaging and analysis.

### Calculations and statistical analyses

Proportions and absolute numbers of MDMs (non-infected or infected with live/dead LD-BCG) were directly deduced from flow cytometry analyses. Absolute numbers could be compared since equal sample volumes were acquired (as defined by count bead normalization, see above). Combinations of different effects (i.e. proportional and absolute reduction of MDM subsets) were then calculated and depicted (examples are shown as Fig 2B, lower right graph and as Fig 3B right graphs). Inter-individual comparisons of cytotoxic efficacy required normalization between different experiments (confounded otherwise by different infection efficacy and MDM/effector cell viability). We therefore set the numbers of MDM infected with LD-BCG without effector cells to 1 (≈100%) and calculated relative differences of samples co-cultured with different effector cells and E/M ratios. This value is termed ‘cytotoxic efficacy’ throughout the manuscript.

Statistical calculations were performed using GraphPad Prism 6 (GraphPad Software). Non-parametric Mann-Whitney U-test was chosen for cytotoxic efficacy comparisons of different effector cell stimuli and proportions of early and late apoptotic markers expressing MDMs. The two-way ANOVA test was applied for comparisons of effector cell titration-dependent cytotoxic efficacy. *P*-values < 0.05 were considered to be significantly different.

## Supporting information

S1 Fig Gating procedure of flow cytometry analyses for MDM infected with LD-BCG and co-cultured effector T cells. Arrows indicate the sequence of analyses to determine apoptosis markers 7-AAD and Annexin-V on MDM as well as lineage marker CD4 and CD8 on CD3 positive T cells.
(PDF)
