# Supplementary material for: A novel mycobacterial In Vitro infection assay identifies differences of induced macrophage apoptosis between CD4+ and CD8+ T cells
Source: PLoS One. 2017 Feb 15;12(2):e0171817. doi: 10.1371/journal.pone.0171817 (PMC5310865; doi:10.1371/journal.pone.0171817)
Supplement: S1 Fig — Arrows indicate the sequence of analyses to determine apoptosis markers 7-AAD and Annexin-V on MDM as well as lineage marker CD4 and CD8 on CD3 positive T cells. (PDF) [file pone.0171817.s001.pdf]

Supplementary Figure 1

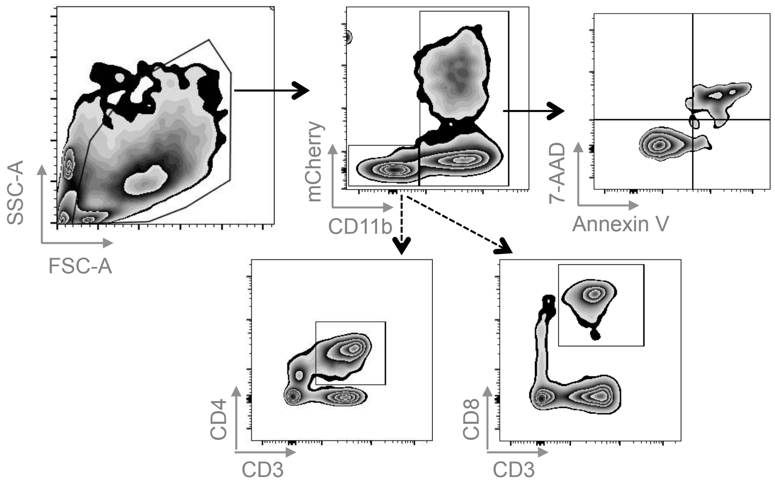

Gating procedure of flow cytometry analyses for MDM infected with LD-BCG and co-cultured effector T cells. Arrows indicate the sequence of analyses to determine apoptosis markers 7-AAD and Annexin V on MDM as well as lineage markers CD4 and CD8 on CD3 positive T cells.
